# Association Between Helicobacter pylori and Gastric Carcinoma

**DOI:** 10.7759/cureus.15165

**Published:** 2021-05-22

**Authors:** Jaskamal Padda, Khizer Khalid, Ayden Charlene Cooper, Gutteridge Jean-Charles

**Affiliations:** 1 Internal Medicine, JC Medical Center, Orlando, USA; 2 Internal Medicine, Advent Health and Orlando Health Hospital/JC Medical Center, Orlando, USA

**Keywords:** helicobacter pylori, gastric carcinoma, caga, vaca, gastric mucosa

## Abstract

Gastric carcinoma is the third leading cause of cancer mortality worldwide. In 2018, the incidence of gastric carcinoma worldwide was over 1,000,000 new cases, with approximately 783,000 deaths. The rate of new cases is noticeably increased in Eastern Asia. *Helicobacter pylori* is responsible for the increased incidence of gastric cancer. In the year 2015, *H. pylori* had an approximate prevalence of 4.4 billion positive cases worldwide, with the most positive cases found within the region of Africa, Latin America and the Caribbean, and of Asia. *H. pylori* is known to have multiple strains which allow it to survive in the host cell epithelium chronically. Research has shown many factors which play a significant role in developing infection and thereafter its progression to gastric carcinoma. After *H. pylori* colonizes the gastric mucosa, its effects can be potentiated by virulence factors, host factors, and environmental factors. *H. pylori* contains virulence factors that aid in the adhesion, translocation, inflammation, and infectivity of the host gastric epithelium. It alters the functions of the host immune response and cytokines, utilizing these factors to invade and persist in the gastric epithelium for a long period of time. The human body will identify *H. pylori* to be foreign and will exacerbate an inflammatory response in an effort to eradicate the bacterium. Consequently, this will cause *H. pylori* to induce a serious infection which may progress to cancer. In this review, we will discuss the various factors involved in the infectious process of *H. pylori* and how they help the infection progress to gastric carcinoma. This will allow us to better understand and modulate treatments to effectively eradicate this bacterium before it triggers the body into developing cancer.

## Introduction and background

**Figure 1:**
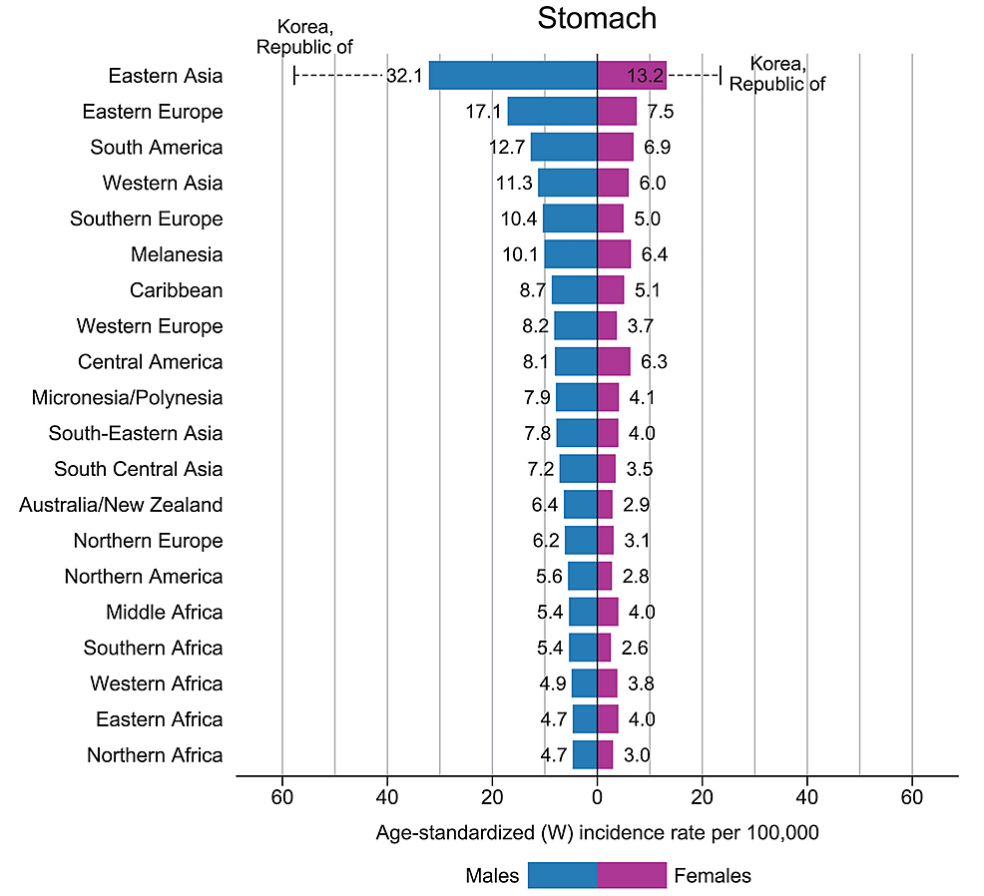
The Year 2018 Bar Graph of Region‐Specific Incidence Age‐Standardized Rates by Gender for Gastric Carcinoma. Rates of stomach cancer shown in descending order across the world (W). This figure was originally published in the American Cancer Society Journals [2]. Permission to reproduce/reuse this figure was obtained from John Wiley and Sons on May 08, 2021. License number: 5064360986878.

American Cancer Society (ACS) estimates the incidence and mortality of gastric carcinoma on a yearly basis within the United States. For the year 2021, ACS has estimated about 26,560 cases of gastric carcinoma will be diagnosed. Of those cases, 16,160 are men and 10,400 are women. The mortality rate due to gastric carcinoma is estimated to be approximately 11,180 deaths, 6,740 occurring in men and 4,440 occurring in women [1]. Gastric carcinoma (cardia and non-cardia gastric carcinoma combined) is considered the third leading cause of cancer mortality, and the fifth leading cause of malignancy worldwide. In 2018, the incidence of gastric carcinoma worldwide was over 1,000,000 new cases, with approximately 783,000 deaths. The rate of new cases is noticeably increased in Eastern Asia (Figure 1) [2]. A major cause of gastric carcinoma is attributed to *Helicobacter pylori*, which accounts for 90% of non-cardia gastric carcinoma [2]. In the year 2015, *H. pylori* had approximately a prevalence of 4.4 billion positive cases worldwide, with the most positive cases within the region of Africa, Latin America and the Caribbean, and Asia. Africa has shown to have higher cases of *H. pylori*, but the rate of gastric carcinoma is still lower compared to China or Japan. This was thought to be due to the *H. pylori* strain lacking cytotoxin-associated gene pathogenicity island (cag PAI) [3]. *H. pylori* is a flagellated, spiral-shaped, Gram-negative bacteria that enters and colonizes the stomach. It can survive in the acidic environment with the help of its virulence factors. *H. pylori* can cause various conditions such as inflammation of the stomach lining, leading to gastritis. It can increase the risk of developing ulcers and potentially gastric carcinoma [4]. Strong correlations have been found between *H. pylori* and gastric carcinoma. The significant risk of developing gastric carcinoma due to *H. pylori* infection can be determined by reviewing evidence-based research identifying impactful virulence factors, host immunity, and environmental factors.

## Review

Virulence factors

Cytotoxin-Associated Gene

The cag PAI encodes one of the strongest virulence factors of *H. pylori*, cytotoxin-associated gene (CagA) [5]. CagA was identified in the early 1990s and is closely associated with peptic ulceration and gastric carcinoma. Strains of *H. pylori* that contain cag PAI increase the risk for gastritis and gastric carcinoma compared to those who lack cag PAI [5]. *H. pylori* facilitates its infection with bacterial attachment/adhesion to the host gastric epithelium. This is accomplished with the help of adhesins and outer membrane proteins (OMP) [5]. Once the bacterial attachment is established, the type 4 secretion system (T4SS) is required to gain entry into the host epithelium. Proteins such as CagA, CagL, and CagY are required for the formation of the T4SS. It appears to be a syringe-like pilus structure that ultimately injects CagA into the host epithelium [5]. Various proteins are required by T4SS for the translocation of CagA into the host gastric epithelial cells. Host cell stabilization occurs by T4SS proteins CagY and CagL interaction with a5β1. Along with cell stabilization, CagL also facilitates the secretion of CagA into the host cell [6]. Epithelial cells have carcinoembryonic antigen-related cell adhesion molecule (CEACAM) protein receptors which bind to bacterial HopQ for translocation of CagA into gastric epithelial cells along with the release of pro-inflammatory mediators. *H. pylori* also stimulates the host cell plasma membrane to expose its membrane phospholipid, phosphatidylserine, so that CagA can interact with it and initiate its secretion into the cell [6].

Once inside the cell, CagA is phosphorylated at its (Glu-Pro-Ile-Tyr-Ala) EPIYA motifs located in the carboxy-terminal by tyrosine-protein kinase Src (c-Src) and tyrosine-protein kinase Abl (ABL1). After phosphorylation and translocation of CagA in the human gastric adenocarcinoma cell line, there is a morphological transition at a cellular level causing cell elongation and scattering. This is known as the hummingbird phenotype [6]. The phosphorylated CagA causes activation of host cell tyrosine kinase (SHP-2) along with many other intercellular activators. This causes persistent activation of extracellular signal-regulated kinases 1 and 2 (ERK 1 & 2) and C-terminal src kinase (Csk) [7]. In the human gastric adenocarcinoma cell line, the CagA-SHP2 complex results in cell elongation by increasing the period of ERK activation and by dephosphorylating focal adhesion kinase (FAK) [8]. FAK is involved in cell adhesion and cell migration processes. Cell elongation also results from phosphorylated CagA inhibiting the catalytic function of c-Src, leading to the dephosphorylation of actin-binding proteins [9]. Phosphorylated CagA interacts with the SHP-2 protein, which then inhibits protease-activated receptor-1/microtubule affinity regulating kinase (PAR1/MARK kinase), further compromising epithelial tight junctions. PAR1/MARK kinase is known to preserve gastric epithelial polarity and junctional integrity [10]. CagA phosphorylation results in many outcomes such as reorganization of the actin cytoskeleton, motility, and activation of inflammatory cells, increased cell proliferation, and mitogenic gene transcription [11].

These results are also seen with the interaction of unphosphorylated CagA with a number of intercellular proteins such as zonulin-1 (ZO-1), occludin, junctional adhesion molecules (JAM), and other elements of adherens and tight junctions of gastric epithelial cells. By disrupting the adherens and tight junctions, *H. pylori* is able to obtain access to epidermal growth factor receptors (EGFR) and Her2/Neu on the basolateral membranes, which may facilitate cell damage and mucosal ulceration. It may also allow *H. pylori* access to mucosal immune cells [12].

Vacuolation Cytotoxin A

Vacuolation cytotoxin A (VacA) causes vacuole formation leading to loss of gastric epithelial cell integrity, along with inhibition of T-cell proliferation, promotion of mitochondrial damage, and apoptosis. These functions permit *H. pylori* to persist as a chronic infection by evading the immune system.

*H. pylori* T4SS secretes VacA, which is a major toxin protein, that once inside the cell will lead to vacuolation. This is theorized to be caused by VacA anion-selective channels within the vesicular membranes. These channels cause increased intraluminal chloride concentrations from increased Cl- ion transport, overall leading to osmotic swelling and vacuole formation in the cytoplasm of gastric cells. VacA also obstructs T-cell proliferation, interrupts mitochondrial function, and triggers apoptosis. It is presumed that VacA is capable of causing a reduction of the mitochondrial electrochemical membrane potential by forming pores at the inner mitochondrial membrane which potentiates the release of cytochrome c and causes cell death. The exact mechanism is unclear on how VacA induces apoptosis [13].

VacA inhibits the activation of T cells through T-cell receptor (TCR) and CD28 interaction. VacA is also capable of inhibiting T-cell proliferation in the presence of IL-2 and without the help of nuclear factors of activated T cells (NFTA). Altogether this allows *H. pylori* to effectively evade the immune system and persist as a chronic infection [14].

High-Temperature Requirement A

*H. pylori* secretes a virulence factor known as high-temperature requirement A (HtrA), which has two purposes; it functions as a chaperone and as a serine protease. *H. pylori*-secreted HtrA cleaves E-cadherins which are located on epithelial cells, leading to cell junction destruction. This disrupts the gastric epithelial barrier, therefore, allowing access to *H. pylori* [15]. Chaperone functions of HtrA are required for protein control. Misfolded proteins are refolded or degraded by HtrA. This overall allows for bacterial growth and survival under stressful conditions [16].

Blood Group Antigen-Binding Adhesin

Blood group antigen-binding adhesin (BabA) is an outer membrane protein of *H. pylori* which is recognized by Lewis b blood group antigens (Leb) located on host gastric epithelial cells. *H. pylori* strains expressing BabA are more severe and have higher bacterial colonization ability [17]. While three alleles of Bab (BabA1, BabA2, and BabB) have been discovered, only the BabA2 genotype is required for binding to Leb [18]. Translocation of CagA into the host cell via T4SS is mediated by BabA binding to host epithelial cells [19]. The severity of *H. pylori* complications is worse with the expression of CagA, VacA, BabA2, as these virulence factors work concurrently. This will lead to more aggressive inflammation and a greater risk for gastric carcinoma [18].

Sialic Acid-Binding Adhesin

Sialic acid-binding adhesin (SabA) is an *H. pylori* adhesin that binds to the Sialyl-Lewis X antigen which is found on the gastric epithelium. There is increased expression of Sialyl-Lewis X during an inflammatory response by the host, which allows for increased adhesion of *H. pylori* to the gastric mucosa with the help of SabA [20]. SabA has been proven to be involved in the progression of gastric disease, gastric atrophy, and gastric carcinoma [20]. SabA also has the capability to mimic selectin in order to activate host neutrophils. This leads to the production of reactive oxygen species (ROS), which contributes to the persistent inflammatory response [21].

Gamma-Glutamyl Transpeptidase

Gamma-glutamyl transpeptidase (GGT) has been proven to have many functions. It promotes the colonization of *H. pylori* in the gastric mucosa, stimulates apoptosis of gastric epithelial cells, and causes inhibition of T-cell proliferation and differentiation [22]. GGT is located in the outer membrane vesicles of *H. pylori*. It has been associated with elevated levels of hydrogen peroxide and Il-8 production in the gastric epithelial cells [23]. GGT causes the depletion of glutamate and glutathione, increasing the production of ROS and the production of ammonia. These products allow *H. pylori* to cause cell-cycle arrest, apoptosis, and necrosis in epithelial cells, further resulting in cancer [22].

Outer Inflammatory Protein A

Outer inflammatory protein A (OipA) is an inflammatory-related outer membrane protein, which is encoded by the HopH gene. A more significant inflammatory response is produced by OipA positive *H. pylori* strains compared to OipA negative strains. This leads to a greater risk of gastric carcinoma and gastric ulcer disease [24]. OipA is more widely found in the gastric biopsy samples acquired from patients with precancerous gastric lesions compared to those with only gastritis [25]. The binding of OipA to the gastric cells is also associated with the activation of the apoptotic cascade through the Bcl-2 pathway [26]. OipA is known to promote the secretion of numerous proinflammatory cytokines such as Il-1, Il-6, and Il-8. It also inhibits the release of IL-10 and dendritic cell maturation, therefore, increasing the risk of gastric carcinoma [27].

Host factors

Tumor Necrosis Factor-a

Tumor necrosis factor-a (TNF-a) is a pro-inflammatory cytokine that is elevated in *H. pylori* infection. It inhibits gastric acid secretion, thus allowing *H. pylori* to survive and spread [28]. TNF-a plays an important role in gastric carcinoma through the activation of the Wnt/β​​​​​​-catenin signaling pathway [29]. The Wnt pathway has been known to be associated with promoting cancer growth and embryonic development. It controls embryonic cell proliferation, cell migration, body axis patterning, and cell fate specification for the proper formation of important body tissues [30]. Oguma et al. have identified a connection between elevated nuclear accumulation of b-catenin and macrophage infiltrated gastric mucosa. *H. pylori* infection causes an inflammatory reaction involving macrophage accumulation. Macrophages release TNF-a which promotes the upregulation of the Wnt/β​​​​​​-catenin signaling, therefore contributing to gastric carcinoma development [31].

Interleukin-1 β

Interleukin-1 β (IL-1β) is a pro-inflammatory genotype of IL-1. Expression IL-1β is alleged to inhibit the secretion of gastric acid and cause hypochlorhydria. It is capable of inhibiting acid secretion more potently compared to proton pump inhibitors and H2 blockers. This inhibition of acid secretion allows for *H. pylori* colonization, leading to inflammatory mucosal damage and increased risk of gastric carcinoma [32].

Interleukin-8

Interleukin-8 (IL-8) is a potent chemokine secreted by gastric epithelial cells in response to *H. pylori*, which has a direct association with gastric carcinoma. Higher levels of IL-8 are correlated to a poorer outcome and a more aggressive gastric carcinoma. IL-8 binds to C-X-C motif chemokine receptors 1 and 2 (CXCR1 and CXCR2) on endothelial cells. This causes proliferation, migration, and survival of endothelial cells; it causes angiogenesis and enhances cancer survival. The binding of IL-8 to CXCR2 causes vascular endothelial growth factor (VEGF) expression leading to the formation of new blood vessels. Overexpression of the cell surface receptors CXCR1 and CXCR2 can facilitate migration and invasion of cancer cells through the basement membrane. IL-8 is capable of disrupting the basement membrane by the secretion of metalloproteinase, aiding in invasion and metastasis [33]. IL-8-induced angiogenesis and invasion are involved in metastasis of gastric carcinoma [33].

Interleukin-10

Interleukin-10 (Il-10) is an anti-inflammatory cytokine whose function is to decrease the cell-mediated immune response and cytotoxic inflammatory response. A study by El-Omar et al. reported that the presence of Il-10 may more than double the risk of having gastric carcinoma [28]. Another study by Sánchez-Zauco et al. suggested that gastric carcinoma produces its own Il-10 which functions to block anticancer responses. This explains why Il-10 levels decrease once the cancer is removed [34]. Teymournejad et al. reported that OpiA suppresses the release of IL-10 leading to an increased risk of gastric carcinoma [27]. These findings demonstrate the potential for further research that needs to be conducted on how exactly IL-10 plays a role in gastric carcinoma.

Nuclear Factor Kappa B

Nuclear factor kappa B (NFkB) is a transcription factor that regulates genes involved in the proliferation of cells, inflammation, and apoptosis [35]. In normal conditions, NFkB is found in the cytoplasm and is inhibited by IkB-molecules. In stimulatory conditions, IkBs are degraded which leaves NFkB free to translocate to the nucleus and exert its effects [36]. Inflammatory conditions as well as cancer is able to modify the activity of enzymes that control NFkB function [35]. *H. pylori* is able to produce a number of virulence factors (e.g., cagPAI, CagA, VacA) that are able to stimulate the NFkB pathway. The stimulation of this pathway leads to the accumulation of inflammatory cells and mediators that promote gastric carcinoma [37]. *H. pylori* strains which are cagPAI-positive are more potent inducers of NFkB activity [36]. NFkB plays a role in regulating proliferation, angiogenesis, and evasion of apoptosis. NFkB causes angiogenesis byways of VEGF and evades apoptosis by increased expression of anti-apoptotic genes such as Bcl-2 and Bcl-xL. It also promotes cell proliferation by increasing the expression of COX-2 [36,37]. Studies have hypothesized that pharmacological inhibition of NFkB can be promising in anticancer therapies by promoting cell death [36].

Cyclooxygenase-2

Overexpression of cyclooxygenase-2 (COX-2) has been associated with the growth and progression of gastric carcinoma. COX-2 is the rate-limiting enzyme in the production of prostaglandins. Through the COX-2/prostaglandin E2 (PGE2) pathway there is an increased amount of PGE2 produced, which is the prostaglandin involved in neoplasm formation. COX-2 contributes to the development of gastric carcinoma by inhibition of apoptosis, stimulating cell proliferation, angiogenesis, invasion, and metastasis. Upregulation of COX-2 is an inflammatory response to the presence of *H. pylori*, which can stimulate increased amounts of PGE2. COX-2 has activator protein 2 (AP-2) and NFkB binding sites. It is presumed that NFkB activation upregulates COX-2 levels, which can further decrease the anti-proliferative effects of transforming growth factor-beta (TGF-b). COX/PGE2 pathway can induce the production of VEGF to stimulate the growth of blood vessels. E-cadherin normally functions to avert the invasion of cancer by obstructing cancer cell separation from tissue. E-cadherin is lowered by Cox-2 leading to invasion and metastasis [38].

P53

P53 is a tumor suppressor gene that regulates the cell cycle and potentiates tumor suppression [39]. Inactivation of P53 is an important step in the development of gastric carcinoma and is seen in about 40% of gastric tumors. P53 is downregulated by the human double minute 2 (HDM2) protein. *H. pylori* activates serine/threonine kinase (AKT kinase) in the infected gastric cells, which then goes to phosphorylate HDM2. HDM2 can now decrease the P53 in the gastric mucosa [40]. CagA is also able to directly induce phosphorylation of HDM2, causing decreased P53, as well as increasing the survival of DNA-damaged cells [39,40]. It has also been reported that *H. pylori* is able to cause mutation of P53 by inducing cytidine deaminase expression [40]. Therefore, the mutation in p53 may play a role in gastric carcinoma and may also serve as a unique biomarker and treatment target for gastric carcinoma [41].

ROS

ROS are produced by many host cells such as neutrophils and epithelial cells in response to *H. pylori*. ROS production within the neutrophil occurs in an attempt to eliminate the bacteria by NADPH oxidase. Neutrophils are located within the tissue and the pathogen is located in the lumen which makes eradication of *H. pylori* challenging. This leads to chronic infection causing inflammation, oxidative stress, and damage to gastric mucosa eventually resulting in gastric carcinoma. Epithelial cells are also able to produce a small amount of ROS since they also have an NADPH subunit Nox. Many virulence factors of *H. pylori* can cause oxidative stress, such as CagA, VacA, SabA, and GGT. *H. pylori* stimulates the release of neutrophils from the host cells which attempt to eradicate the pathogen. However, *H. pylori* is able to evade it leading to chronic inflammation and infection, resulting in harm to the host. This can result in DNA damage, prevent DNA repair, overall leading to cancer [42].

Environmental factors

Dietary Salt

Intake of high levels of dietary salt has been connected to the development of gastric carcinoma. In a study by Gaddy et al, using Mongolian gerbils, there was upregulation of CagA in response to high salt conditions. They were maintained on either a regular or high salt diet with the CagA positive *H. pylori* infection. The latter group exhibited a higher rate of inflammation and gastric carcinoma, with increased CagA transcription [43]. One or two copies of the DNA motif TAATGA were present upon analysis of the promotor region in CagA. At least two copies of the TAATGA motif were necessary for the upregulation of CagA, by elevated dietary salt. However, little to no increase in CagA expression was observed with one copy of TAATGA motif [44].

Cigarette Smoking

Cigarette smoking interaction with CagA seems to play a role in *H. pylori-*induced gastric carcinoma [45]. Active smokers showed an increased risk of colonization of the gastric mucosa with CagA virulence factor compared to non-smokers [46]. Smoking was found to increase the risk of gastric carcinoma in a moderate amount, but the risk for gastric carcinoma with the combination of smoking and CagA positive strain produced a greater effect [45]. The risk of gastric carcinoma was higher when both factors were present, demonstrating that there are collaborative effects between smoking and CagA in gastric carcinoma [45,46].

Diagnosis

H. pylori Diagnosis

Early diagnosis of *H. pylori* is crucial to allow for faster detection and treatment, preventing further damage and gastric pathologies. *H. pylori* can be diagnosed by non-invasive testing such as the urea breath test, stool/fecal antigen testing and *H. pylori* antibody testing, or by invasive tests such as endoscopy with biopsy [47].

H. pylori Urea Breath Test

The most commonly used tests are the urea breath test and stool antigen testing since they are quick and non-invasive. For the urea breath test, the patient is given a urea liquid to drink. Breath samples are collected and examined before and after ingestion of the harmless radioactive material. In the presence of *H. pylori,* urea is broken down into labeled carbon dioxide (CO_2_). The lab will inspect if labeled CO_2_ is higher in the latter exhaled sample, which will confirm the presence of *H. pylori* [47].

H. pylori Stool Antigen and Antibody Test

Stool/fecal antigen test is another non-invasive test that detects *H. pylori* antigens within the stool. Diagnosis of the bacterium can also be done by *H. pylori* antibody testing. This test is able to detect antibodies to the pathogen, but it will not be able to differentiate whether it is a current or old infection. A positive *H. pylori* antibody testing should be followed up by a urea breath test or stool/fecal antigen test [47].

H. pylori Endoscopy With Biopsy

A more invasive test is endoscopy with biopsy. This test is not used as much due to its invasiveness. In an endoscopy, tissue samples are obtained and sent to be examined under a microscope to identify *H. pylori* bacteria. The presence of urease in a tissue sample can also be helpful since *H. pylori* is known to generate ureases to help it survive in the stomach. This test is known as rapid urease testing. Cultures can also be done, where the bacteria require weeks to grow in a media to determine antibiotic susceptibility. Polymerase chain reaction (PCR) identifies the pathogen by amplifying DNA [47].

Gastric Cancer Diagnosis 

If *H. pylori* has progressed and gastric carcinoma is suspected, then confirmation is required. The following tests and procedures can be used along with physical examination findings for the diagnoses. Non-invasive imaging studies such as X-ray, CT scan, magnetic resonance imaging (MRI), positron emission tomography (PET) scan to more invasive tests such as upper endoscopy with biopsy or laparoscopy can be used to confirm the presence of gastric carcinoma [48].

Upper Endoscopy With Biopsy 

The preferred test is an upper endoscopy, allowing the doctor to see the inner lining of the upper gastrointestinal tract with a thin flexible tube that has a camera on its end. During the procedure, a small tissue sample biopsy can be taken for microscopic examination. During the microscopic examination, the sample is checked for cancer, and if found, lab tests for genes and proteins are done for possible treatment. Imaging studies can be done to see if cancer is present, to learn about the depth of cancer, and to see if treatment is working [48].

Upper GI Series

The upper GI series is not used as much as an upper endoscopy anymore since it can fail to see abnormal areas and does not allow for biopsy. Upper GI series is done by consuming barium, which coats the lining of the GI, therefore outlining and showing abnormalities if present [48].

Imaging: CT, PET, MRI

A CT can verify the location of neoplasm along with metastasis to nearby organs or lymph nodes. CT scans are also used for CT-guided needle biopsy, where a needle is placed into the mass and a sample is removed for lab testing. MRI may also be helpful, but it is not used as much as a CT. A PET scan is helpful in identifying cancer metastasis. PET scan is not as detailed as CT or MRI, but it is able to examine the whole body at once to look for metastasis. In this test, a radioactive sugar is injected into the patient. Since cancer cells are more active, they will absorb the radioactive sugar leading to the discovery of the spread. Endoscopic ultrasound is often used to understand the depth of the spread into the gastric wall or lymph nodes. An ultrasound probe is positioned on the end of an endoscope to use sound waves to get a better image. Laparoscopy is a surgical procedure done under anesthesia, where a laparoscope is inserted into the abdomen allowing the surgeon to look at the internal organs and lymph nodes and allowing for biopsy if needed. This is usually done after a CT or PET scan has confirmed there is no spread of the neoplasm as of yet, signifying surgery is still a viable option. At this time, a peritoneal wash can also be performed where saline is used and collected in the abdomen to check for cancer cells [48].

Treatment for gastric cancer

There are many different types of treatment options available such as surgery, chemotherapy, radiation, targeted therapy, and palliative care. The choice of treatment must take many factors into account including the location of cancer, depth of spread, patient’s health, and personal preference [49,50].

Surgery

Surgery is an operative procedure where cancer along with healthy tissue is removed, depending on the cancer stage. In the early stage, where cancer is localized to the gastric mucosa, removal of the tumor can be done by endoscopic mucosal resection. This is when an endoscope is used to remove cancer from the stomach lining. In the early stage, when the cancer is localized to the stomach, the surgeon will perform a partial or subtotal gastrectomy. This is a procedure where a portion of the stomach with cancer is excised. In a total gastrectomy, the whole stomach is removed, and the esophagus is connected to the small intestine. Total gastrectomy is used when the cancer is localized to the body of the stomach or the gastroesophageal junction. Lymphadenectomy is often done in conjunction with surgery to determine if cancer has spread to the lymph nodes. Surgery is not recommended when metastasis is involved [49,50]. 

Radiation and Chemotherapy

Chemotherapy is treatment with drugs, while radiation is the use of high-powered beams to destroy cancerous cells, which further inhibits these cells from growing, dividing, and replicating. Chemotherapy drugs will go through the body in an attempt to kill cancer cells that have metastasized outside the stomach. These drugs may be used alongside radiation or targeted drug therapy. Radiation and chemotherapy can be used before or after surgery. Before surgery, it is used to decrease the size of cancer for easier removal, while after surgery it is used to destroy any cancer cells that persist [49,50]. Targeted therapy is when drugs are directed at cancer genes or proteins which help cancer grow and survive. Targeted therapy for gastric carcinoma includes human epidermal growth factor receptor 2 (HER2) and anti-angiogenesis. Research continues to find more specific targets in an attempt to direct new therapies toward them [50].

Immunotherapy 

Immunotherapy helps the immune system fight against cancer by increasing host immunity. Cancer cells make it difficult for the host immune system to be able to fight it by releasing proteins. Immunotherapy is used to block this and restore the host immune system [49,50].

Palliative Care

Palliative care is a supportive treatment focused on working with the patient and their family while undergoing treatment such as surgery, chemotherapy, or radiation. It comprises nutritional, emotional, and spiritual support. Patients who start palliative care alongside treatment have better outcomes and essentially improvement in their quality of life, fewer symptoms, and more satisfaction with treatment [49,50].

## Conclusions

Gastric carcinoma is the fifth leading cause of malignancy worldwide, with a major attribution to *H. pylori*. *H. pylori* is a Gram-negative pathogen capable of invading and colonizing the gastric mucosa, which can also cause various gastric complications ranging from gastritis to gastric carcinoma. This bacterium has many virulence factors that aid and assist in adherence and translocation of *H. pylori* into gastric epithelial cells. The host immune system also releases cytokines and chemokines in response to this pathogen which can potentiate the development of gastric carcinoma. Furthermore, it has been indicated that environmental factors can play a role to enhance the effects of *H. pylori* in gastric carcinoma. High dietary intake of salt and cigarette smoking are important modifiable factors associated with gastric pathology. Early diagnosis and treatment of *H. pylori* are important because of the possibility of progression to gastric carcinoma. If gastric carcinoma is identified, treatment is imperative and can include surgery, chemotherapy, radiation, targeted therapy, and palliative care. Targeted therapy can be aimed towards virulence factors in an attempt to eradicate cancer. Immunotherapy supports the host cells to deliver an effective immune response to *H. pylori* infection, preventing the progression of infection. Further investigation is required to develop an effective treatment of *H. pylori*-induced gastric carcinoma to reduce the overall mortality rate.
